# Development of an Indirect ELISA for the Detection of SARS-CoV-2 Antibodies in Cats

**DOI:** 10.3389/fvets.2022.864884

**Published:** 2022-06-10

**Authors:** Dashzeveg Bold, Gleyder Roman-Sosa, Natasha N. Gaudreault, Batsukh Zayat, Roman M. Pogranichniy, Juergen A. Richt

**Affiliations:** ^1^Department of Diagnostic Medicine/Pathobiology, Kansas State University, Manhattan, KS, United States; ^2^Institut für Virologie, Fachbereich Veterinärmedizin, Justus-Liebig-Universität Giessen, Giessen, Germany; ^3^Institute of Veterinary Medicine, Mongolian University of Life Sciences, Ulaanbaatar, Mongolia; ^4^Veterinary Diagnostic Laboratory, Department of Diagnostic Medicine/Pathobiology, Kansas State University, Manhattan, KS, United States

**Keywords:** COVID-19, SARS-CoV-2, diagnostic, serology, antibodies, ELISA, feline, cat

## Abstract

Companion animals are susceptible to a variety of coronaviruses, and recent studies show that felines are highly susceptible to SARS-CoV-2 infection. RT-PCR diagnostic is currently the method of choice to detect the presence of SARS-CoV-2-specific viral nucleic acids in animal samples during an active infection; however, serological assays are critical to determine whether animals were exposed to the virus and to determine the seroprevalence of SARS-CoV-2-specific antibodies in a defined population. In this study, we utilized recombinant nucleocapsid (N) protein and the receptor-binding domain (RBD) of the spike protein of SARS-CoV-2 expressed in E. coli (N) and mammalian cells (N, RBD) to develop indirect ELISA (iELISA) tests using well-characterized SARS-CoV-2-positive and -negative cat serum panels from previous experimental cat challenge studies. The optimal conditions for the iELISA tests were established based on checkerboard dilutions of antigens and antibodies. The diagnostic sensitivity for the detection of feline antibodies specific for the N or RBD proteins of the iELISA tests was between 93.3 and 97.8%, respectively, and the diagnostic specificity 95.5%. The iELISAs developed here can be used for high-throughput screening of cat sera for both antigens. The presence of SARS-CoV-2-specific antibodies in a BSL-2 biocontainment environment, unlike virus neutralization tests with live virus which have to be performed in BSL-3 laboratories.

## Introduction

The ongoing pandemic of coronavirus disease 2019 (COVID-19) is caused by severe acute respiratory syndrome coronavirus-2 (SARS-CoV-2), a member of the *Coronaviridae* family in the betacoronavirus genus (1). SARS-CoV-2 is an enveloped, single-stranded, positive-sense RNA virus with a large genome size of ~30 kilobases (kb). The family of *Coronaviridae* is divided into four genera: alphacoronavirus, betacoronavirus, deltacoronavirus, and gammacoronavirus (1). Companion animals are susceptible to a variety of coronaviruses: felines can be infected by feline enteric coronavirus (FECV) and feline infectious peritonitis virus (FIPV or referred to here as FeCoV) and canines by canine coronavirus (CCoV) (2, 3), with all these viruses belonging to the alphacoronavirus genus (4). Coronaviruses encode four structural proteins: three are membrane associated (the spike, envelope, and membrane proteins) and one, the nucleocapsid (N) protein, is associated with the viral RNA (5). The spike (S) protein is the major glycoprotein that extends from the surface of the virion forming corona-like spikes (5). The receptor-binding domain (RBD) of the S protein interacts with the cellular receptor angiotensin-converting enzyme II (ACE2) and, therefore, plays a critical role in virus attachment and entry into host cells (5). The RBD is highly immunogenic and the major target of SARS-CoV-2 neutralizing antibodies (6). The N protein packages genomic RNA into the ribonucleoprotein (RNP) complex of the virus; it interacts with the other viral structural proteins, is needed for virus assembly (5), and N-specific antibodies can be detected as early as 8 days post-infection (7).

SARS-CoV-2 was first reported in December 2019 in the city of Wuhan in China (8, 9) and since then spread quickly all over the world. The World Health Organization (WHO) officially declared SARS-CoV-2 a global pandemic on March 11, 2020 (10). According to the WHO, over 446 million human cases and 6 million deaths (as of March 8, 2022) have officially been reported thus far. Although the case fatality rate of SARS-CoV-2 is about approximately 2%, which is lower than for other human sarbecoviruses such as SARS-CoV and Middle East respiratory syndrome (MERS)-CoV, its global spread is causing massive numbers of human cases and deaths and significant economic losses (8).

An accurate diagnosis for SARS-CoV-2 is essential to rapidly quarantine RNA/virus-positive people and to reduce potential virus transmission to naïve individuals (11). RT-PCR is currently utilized as the method to diagnose COVID-19 as recommended by the WHO and CDC (12, 13). It is a highly sensitive and specific method, but inadequate sample collection and technical errors in RNA preparation may produce false-negative results, and cross-contamination can lead to false-positive results. However, shedding of virus or viral RNA is only transient and the RT-PCR test will only be positive during a certain window of time (14).

Serological assays are able to identify SARS-CoV-2-specific antibodies in clinical samples (such as plasma, serum, and saliva). SARS-CoV-2-specific antibodies can be detected using various methods, such as ELISA, virus neutralization assays, and lateral flow tests. The earliest detection of SARS-CoV-2-specific IgM or IgA isotype antibodies in humans is ~5 days post-infection, whereas IgG isotypes are found later around 10–14 days post-infection (15).

Serological assays can be used to investigate ongoing or retrospective assessments of COVID-19 outbreaks. Sero-surveillance can also be used to study seroconversion after infection or vaccination to determine herd immunity. As discussed above, serological assays should not be used as the method of choice to diagnose COVID-19 but in combination with other assays and methods (16).

Although serological assays are not “fit for purpose” for the diagnosis of SARS-CoV-2 during or early after infection, they are critical to determine the effectiveness of vaccine administrations and herd immunity of populations. They can also be a useful tool in combination with RT-PCR when applied, for example, at least 1 week after the onset of symptoms (17). A combination of a COVID-19 serological test with RT-PCR could improve the diagnostic sensitivity of COVID-19 diagnosis significantly (15, 18).

Recently, we and others have demonstrated that domestic cats are susceptible to SARS-CoV-2 by experimental infection and can readily transmit the virus to naïve cats (19–21). Cats inoculated *via* natural routes are readily infected and shed RNA/virus from nasal, oral, and rectal cavities starting from 1 up to 14 days, with peak RNA/virus shedding occurring within the first 7 days after infection (19–22). Experimentally infected cats also develop virus-specific and neutralizing antibody responses to SARS-CoV-2 (19–25). The aim of the present study was to develop an indirect ELISA (iELISA) test to detect SARS-CoV-2 N- and RBD specific antibodies in felines and analyze the cross-reactivity of recombinant SARS-CoV-2 N and RBD antigens with serum from cats positive for antibodies against feline infectious peritonitis coronavirus (FeCoV).

## Materials and Methods

### Recombinant N and RBD Protein Expression in Mammalian Cells

Nucleotide sequences encoding the nucleocapsid (N) protein and the receptor-binding domain (RBD) of the spike (S) protein of the SARS-CoV-2 isolate Wuhan-Hu-1, GenBank accession # MW036243 and # MT380725, respectively, were used for the establishment of the respective plasmids employed for protein expression. The N and RBD genes, plus two strep tags, were cloned into the mammalian expression vector pCAGGS; and the plasmids were purified using the Qiagen Plasmid Midi Kit (Qiagen, Germantown, MD, USA) (Table 1). The N and RBD recombinant proteins were produced in HEK (human embryo kidney) 293 cells after transfection of these cells with pCAGGS plasmid DNA. Transfected HEK-293 cells were cultured in Dulbecco's modified essential medium (DMEM; Fisher Scientific, Chicago, IL, USA). Supernatants from transfected cells were harvested on day 3 post-transfection by collection and centrifugation of the supernatant at 4,000g for 20 min. Recombinant proteins with strep tags were purified *via* affinity chromatography using Strep-Tactin® (IBA Lifesciences) after lysis of cells under native conditions. Recombinant proteins were dialyzed in a dialysis cassette against phosphate-buffered saline (PBS; pH 7.4, 150 mM NaCl, 4 mM EDTA, 10% glycerol; Dialysis Cassette—Thermo Fisher, Rockford, IL, USA). Each protein was concentrated in Pierce protein concentrators (Fisher Scientific, Rockford, IL, USA) and re-suspended in phosphate-buffered saline (PBS). The amino acid sequences of the N- and C-termini of both proteins are listed in Table 1.

**Table 1:** SARS-CoV-2 recombinant antigens used in this study.

**Antigens**	**Location (start and end sequences)**	**Size (amino acid)**	**Expression system**	**Tag**
RBD protein	PNITNLC../.. VLSFELLHAP	192	Mammalian	Double strep tags, C-terminal
N protein	SDNGPQN../.. SADSTQA	418	Mammalian	Double strep tags, C-terminal
N protein	SDNGPQN../.. SADSTQA	418	*E. coli*	6xHis tag, N-terminal

### Recombinant N Protein Expression in *E. coli*

The plasmid DNA encoding the N protein of SARS-CoV-2 (see below and Table 1) was amplified *via* PCR employing the N-containing pCAGGS plasmid using Phusion High-Fidelity PCR Master Mix Kit (Thermo Fisher Scientific, Rockford, IL, USA). To insert the N gene into the pETite N-His SUMO Vector (Lucigen, Middleton, WI, USA), flanking sequences identical to the vector sequence adjoining the insertion sites were added to the 5′-end of the primers as follows: forward primer 5′-CGCGAACAGATTGGAGGTTCCGATAACGGCCC-3′ and reverse primer 5′-GTGGCGGCCGCTCTATTAGGCCTGTGT AG-3′ (Integrated DNA Technologies IDT, IA, USA); the gene was then amplified by PCR. Briefly, the PCR mixture included 1 μL of the plasmid DNA, 3 μL of betaine solution (Sigma–Aldrich, St. Louis, MO, USA), primers at a final concentration of 0.2 μM each, and ddH_2_O in a total volume of 20 μL. PCR cycling conditions were as follows: initial denaturation step at 98°C for 30 s, 28 cycles with 30 s of denaturation at 98°C, 30 s annealing at 59°C, and 50 s extension at 72°C, followed by a final extension for 10 min at 72°C. Following the amplification of the N ORF by PCR, 1 μL of amplicon (~45 ng) and 2 μL of pETite N-His SUMO vector mix were transformed into chemical competent Top10 cells (Lucigen, Middleton, WI, USA). Colonies were screened for the presence of SARS-CoV-2 N-specific DNA by PCR, and positive colonies were selected and amplified; DNA was isolated using a plasmid isolation kit (Qiagen, Germantown, MD, Cat #: 27106).

The plasmid DNA was transformed into BL21(DE3) bacterial cells (Lucigen, Middleton, WI, USA), and PCR-positive clones were grown in LB buffer with 50 μg/mL of kanamycin (GoldBio, St. Louis, MO, USA). Protein expression was induced with 1 mM IPTG (GoldBio, St. Louis, MO, USA) when cell density reached an OD of 0.8, and then the cells were incubated for 3 h at 37°C on a bacterial shaker. Afterward, the IPTG-induced bacteria were harvested by centrifugation at 6,500 × g for 10 mins. The bacterial cells were lysed by BugBuster protein extraction reagent (Millipore Sigma, Burlington, MA, USA), and soluble protein was isolated by centrifugation of the lysate at 13,000 × g for 20 mins. Protein purification was performed using nickel resin, but the protein yield was low. Therefore, the insoluble recombinant protein was solubilized with serial dialysis of 1.0 M to 0.25 M NaCl in PBS.

### SDS–PAGE

Recombinant proteins were analyzed by SDS–PAGE analysis to determine recombinant protein size and integrity. The recombinant protein (15 μg) was mixed with Tris–glycine SDS sample buffer (Thermo Fisher, Rockford, IL, USA), heated at 70°C for 10 min, and then loaded onto a NuPAGE protein gel (Thermo Fisher, Rockford, IL, USA). Staining and de-staining of the gel were performed with eStain L1C protein staining system (GenScript, Piscataway, NJ, USA).

### Indirect ELISA

Plate wells were coated with 100 ng of the respective protein in 100 μL per well coating buffer (carbonate–bicarbonate buffer, catalog number C3041, Sigma–Aldrich, St. Louis, MO, USA), then covered, and incubated overnight at 4°C. The next day, the plates were washed two times with phosphate-buffered saline [PBS (pH = 7.2–7.6); catalog number P4417, Sigma–Aldrich], blocked with 200 μL per well casein blocking buffer (Sigma–Aldrich, catalog number B6429), and incubated for 1 h at RT. The plates were then washed three times with PBS Tween-20 (PBS-T; 0.5% Tween-20 in PBS). Serum samples were pre-diluted 1:400 in casein blocking buffer; then, 100 μL per well was added to the ELISA plate and incubated for 1 h at RT. The wells were washed three times with PBS-T, and then, 100 μL of HRP-labeled goat anti-feline IgG (H + L) secondary antibody (Thermo Fisher, catalog number A18757), diluted 1:2,500, was added to each well and incubated for 1 h at RT.

After 1 h incubation at RT, plates were washed five times with PBS-T, and 100 μL of TMB ELISA Substrate Solution (Abcam, catalog number ab171525, Cambridge, MA, USA) was added to all wells of the plate. Following incubation at RT for 5 min, the reaction was stopped by adding 100 μL Stop Solution for TMB Substrate (Abcam, catalog number ab171529) to all wells. The OD of the ELISA plates was read at 450 nm on an ELx808 BioTek plate reader (BioTek, Winooski, VT, USA).

FeCoV-negative and SARS-CoV-2-negative cat sera were used as negative controls in the iELISAs to determine the cutoff value for negative cat sera. The average OD 450 nm (OD450) value of the negative control cat sera plus 3 × the standard deviation (SD) was used to calculate cutoff values for each assay. Everything above this cutoff was considered positive (26).

### Indirect Immunofluorescence Tests for SARS-CoV-2 and FeCoV

A commercial immunofluorescence assay (IFA) for detection of antibodies to FeCoV was used in this study (VMRD, #SLD-IFA-FIP2) following the manufacturer's instructions.

For SARS-CoV-2 IFA, Vero E6 cells were plated on 96-well tissue culture plates and infected by adding 200 TCID_50_/well of SARS-CoV-2 virus. The cells were incubated at 37°C and 5% CO_2_ for 48 h, then fixed by adding 100 μL of 80% acetone to each well, and incubated for 10 min at room temperature RT. Afterward, each well was washed with 100 μL of PBS one time, and plates were dried for 15 min in a BSC.

The serum samples were diluted in PBS in two-fold serial dilutions from 1:40 to 1:5,120 in a 96-well plate and incubated for 1 h at 37°C. The monoclonal antibodies were tested using only one dilution of 1:200. FITC-labeled secondary antibodies, anti-cat IgG or goat anti-mouse IgG H&L (Jackson Immuno Research Laboratories, Inc., PA, USA; and Abcam Inc., Cambridge, MA, USA), respectively were added into each well of the plate. After incubation of the plate for 30 mins at 37°C, the plate was washed three times with PBS and evaluated by microscopy.

### Virus Neutralization Test

SARS-CoV-2 and FeCoV neutralizing antibodies in sera were determined using microneutralization assays as previously described (20). Briefly, heat-inactivated serum samples were subjected to two-fold serial dilutions starting at 1:20 and tested in duplicate. Then, 100 TCID_50_ of SARS-CoV-2 or FeCoV virus in 100 μL DMEM culture media was added 1:1 to 100 μL of the sera dilutions and incubated for 1 h at 37°C. The mixture was subsequently cultured on Vero E6 or Vero-TMPRSS2 cells (SARS-CoV-2) or Crandell Feline Kidney cells (FeCoV) in 96-well plates. The neutralizing antibody titer was recorded as the highest serum dilution at which at least 50% of wells showed virus neutralization based on the appearance of CPE observed under a microscope at 48–72 h post-infection.

### Serum Panel and Monoclonal Antibodies

Known SARS-CoV-2-positive cat sera used in this study were obtained during experimental SARS-CoV-2 challenge studies of cats (20, 27); sera positivity was confirmed using a classical virus neutralization (VN) test employing Vero E6 cells as described above. The SARS-CoV-2-infected cats were antibody profile defined/specific pathogen-free (APD/SPF) animals with no detectable antibody titers to feline herpesvirus (rhinotracheitis), feline calicivirus, feline panleukopenia virus, feline coronaviruses, feline immunodeficiency virus, Chlamydia felis, and Toxoplasma gondii (obtained from Marshall BioResources, North Rose, NY, USA). A total of 45 SARS-CoV-2-positive and 22 SARS-CoV-2-negative cat sera and 13 FeCoV-positive and 6 FeCoV-negative cat sera were utilized in this study (Table 2). FeCoV-positive and negative cat sera were provided by the Veterinary Diagnostic Laboratory (VDL) at KSU. The FeCoV-positive serum samples used in this study were collected before 2019, and therefore, cats from which the samples were derived were not exposed to SARS-CoV-2 virus. In addition, four in-house developed monoclonal antibodies (mAbs) specific for the RBD of the SARS-CoV-2 spike protein (28, 29) and a FeCoV N protein-specific mAb (Thermo Fisher, #MA182189, IL, USA) were employed for the tests in this study (**>Tables 6, 7**). For in-house utilized developed SARS-CoV-2 mAbs, 7-week-old Balb/c mice were subcutaneously inoculated with 50 μg of recombinant RBD protein and Freund's incomplete adjuvant and boosted subcutaneously 14 days later. Antibody titers against the RBD antigen were tested within 1 week after the first booster. The mice with the highest antibody titers were selected for additional subcutaneous boosters at 21 days, and an intraperitoneal injection at 28 days. The mice were euthanized 3 days after the last booster, and hybridomas were generated following the fusion of the mouse splenocytes and Sp2/0-Ag14 myeloma cells (ATCC #CRL-1581, Rockville, MD, USA). Screening of desired hybridomas was performed using the recombinant RBD protein using an indirect ELISA, and specificity was confirmed by IFA on SARS-CoV-2-infected cells.

**Table 2:** Cat sera and monoclonal antibodies used in the study.

**Antibodies**	**Number of samples**	**Tests**
SARS-CoV-2-positive cat sera	45	SARS-CoV-2, VNT
SARS-CoV-2-negative cat sera	22	SARS-CoV-2, VNT
FeCoV-positive cat sera	13	IFA, FeCoV
FeCoV-negative cat sera	6	IFA, FeCoV
SARS-CoV-2 RBD-specific mAbs	4	SARS-CoV-2, VNT
FeCoV N-specific mAb	1	IFA, FeCoV

## Results

### Establishment of Indirect ELISA (iELISA) Tests

To determine the optimal amounts of antigen and antibody concentrations in the iELISAs, checkerboard titrations were performed using a range of concentrations (25–400 ng) of the recombinant proteins and two-fold dilutions (starting at 1:25) of a known SARS-CoV-2-positive and negative cat serum [cat serum #026 obtained at−1 day post-challenge (DPC) and at 21 DPC of an experimental SARS-CoV-2 challenge study] (20). We found that coating the wells with 100 ng antigen and using a serum dilution of 1:400 was the optimal combination for the three recombinant antigens tested, based on the best ratio of positive vs. negative serum OD values. The optimal dilution of HRP-labeled secondary anti-cat antibody was estimated with two-fold serial dilutions starting with 1:1,250 up to 1:10,000; the best ratio of OD values for positive and negative cat serum was at a 1:2,500 dilution of the secondary antibody for the three recombinant antigens.

FeCoV-negative and SARS-CoV-2-negative cat sera were used as negative controls in the iELISAs to determine the cutoff value for negative cat sera. For the bacteria-expressed N protein (N_E_), the highest OD450 with the negative cat sera was 0.251 and the lowest OD450 was 0.195. The cutoff value of the *E. coli*-expressed N protein iELISA test was determined to be OD450 0.31; therefore, samples with an OD450 value below the cutoff of 0.31 were considered negative. For the mammalian-expressed N protein (N_M_), the highest OD450 for negative control sera was 0.198 and the lowest OD450 0.108, resulting in a cutoff value of 0.26. The highest and lowest OD450 of the negative cat sera for the mammalian-expressed RBD protein was 0.3 and 0.069, respectively. This resulted in the cutoff value of 0.36 for the RBD iELISA.

### Determination of Diagnostic Sensitivity and Specificity of the iELISA Tests

A total of 45 SARS-CoV-2-positive and 22 SARS-CoV-2-negative cat sera were used to evaluate the diagnostic sensitivity and specificity of the iELISAs employing the three different SARS-CoV-2 antigens, namely, the N protein expressed in *E. coli* and the RBD and N proteins expressed in a mammalian cell expression system.

**Figure 1:**
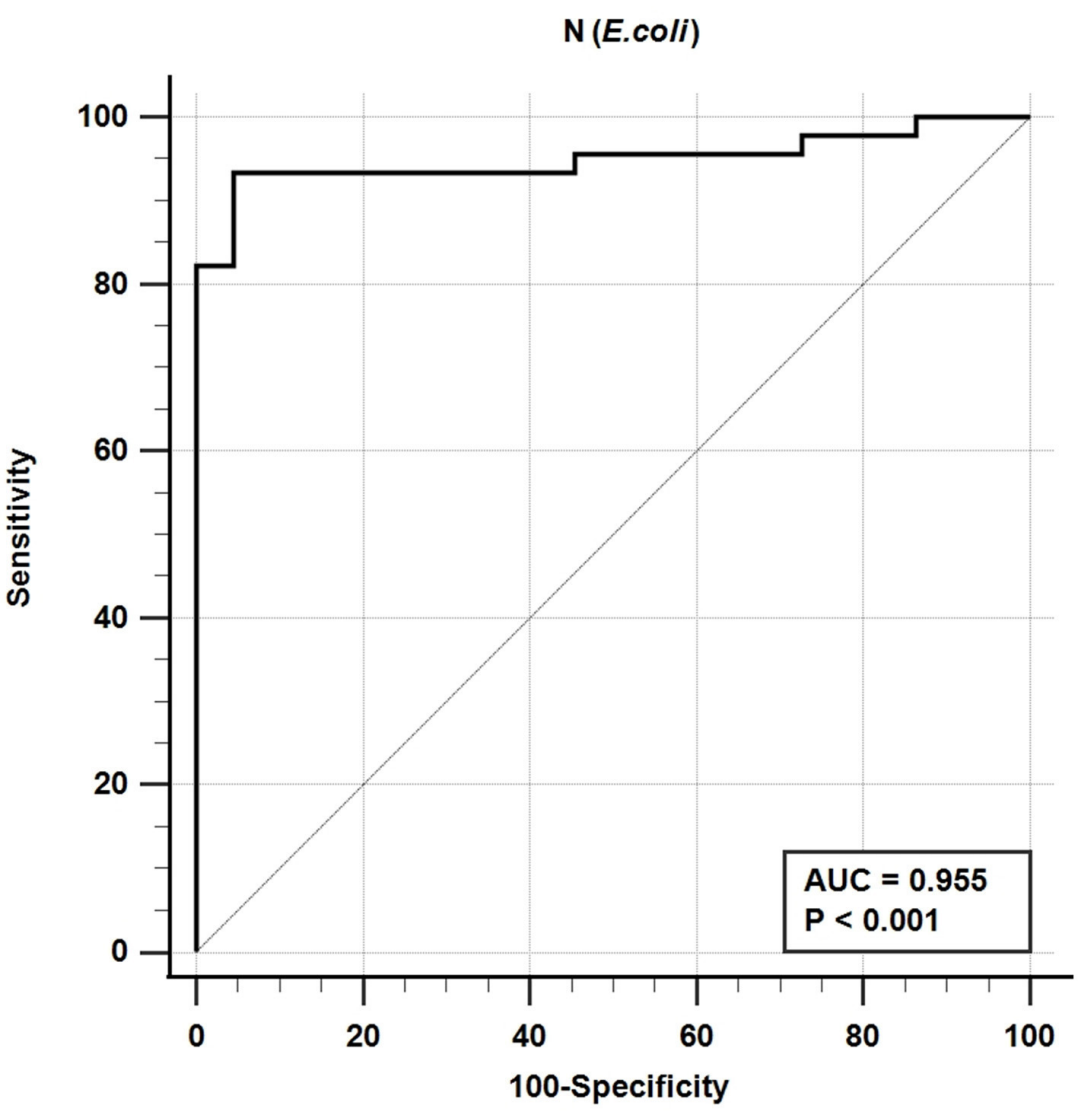
Receiver-operating characteristic (ROC) analysis for the N_E_ protein iELISA. The line represents the 95% confidence interval, and the upper left corner is the highest Youden index indicating the best combined sensitivity and specificity of 93.3% and 95.5%, respectively. AUC, area under the curve.

The iELISA using the recombinant bacteria-expressed N protein and well-characterized cat sera from experimentally SARS-CoV-2-infected cats revealed that 42 out of 45 SARS-CoV-2-positive sera were positive and 21 out of 22 SARS-CoV-2-negative sera were negative. This indicates a diagnostic sensitivity and specificity of the N-specific iELISA of 93.3 and 95.5%, respectively (Table 3; Figure 1).

**Table 3:** Diagnostic sensitivity (Sn) and specificity (Sp) of the iELISA based on *E. coli*-expressed N protein for detecting SARS-CoV-2-specific antibodies in cat serum samples.

**iELISA**	**Positive**	**Negative**	**Total (number)**
Positive (number)	42 **(Sn = 93.3%)**	1	43
Negative (number)	3	21 **(Sp = 95.5%)**	24
Predictive value	97.7%	87.5%	

**Figure 2:**
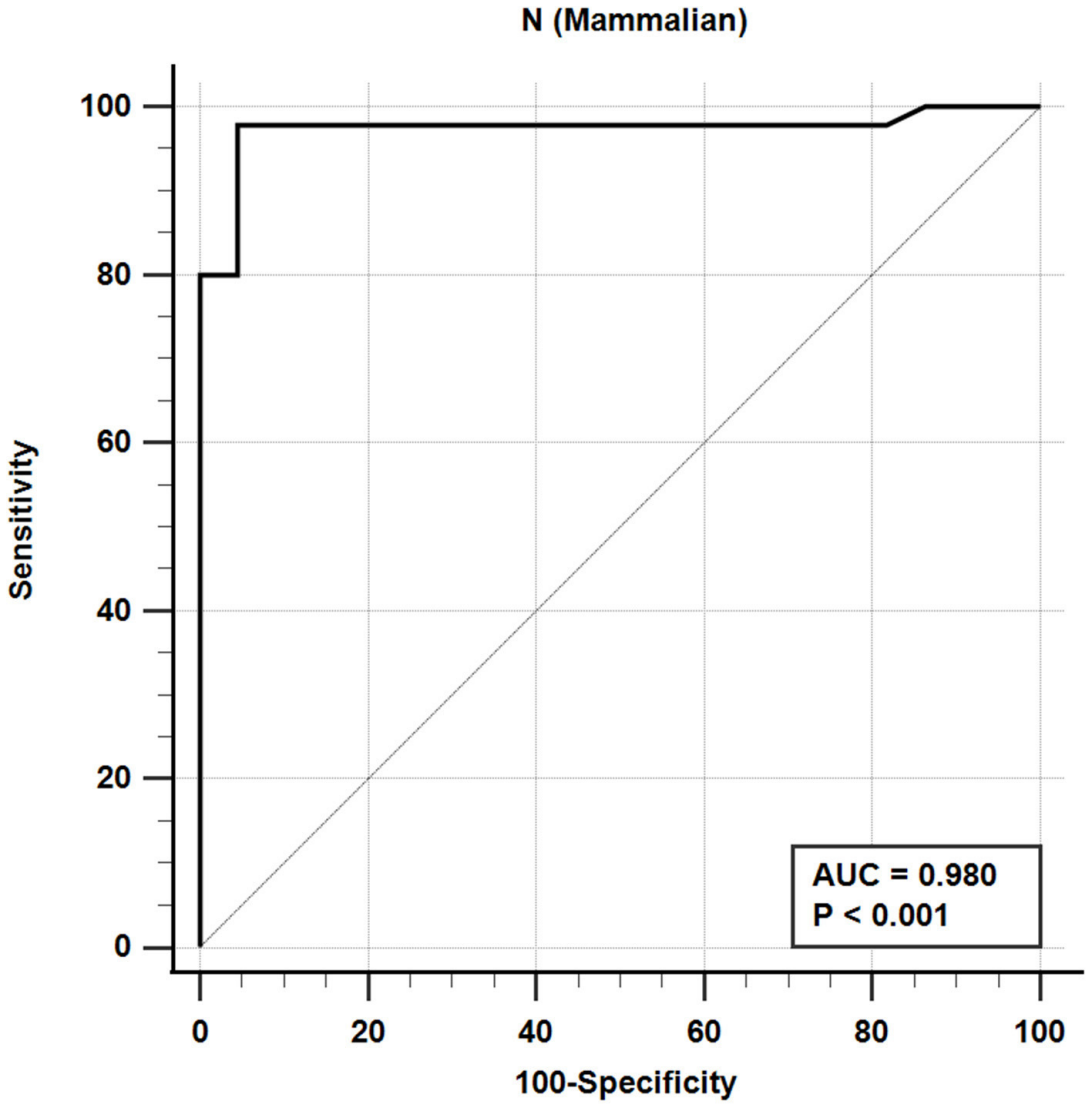
Receiver-operating characteristic (ROC) analysis for the N_M_ protein iELISA. The line represents 95% confidence interval, and the upper left corner is the highest Youden index indicating the best combined sensitivity and specificity of 97.8% and 95.5%, respectively. AUC, area under the curve.

The iELISA using the recombinant mammalian-expressed N protein and well-characterized cat sera from experimentally SARS-CoV-2 virus-infected cats revealed that 21 out of 22 SARS-CoV-2-negative sera were negative and 44 out of 45 SARS-CoV-2-positive sera were positive. This indicates a diagnostic sensitivity and specificity for the mammalian cell-expressed N-specific iELISA of 97.8 and 95.5%, respectively (Table 4; Figure 2).

**Table 4:** Diagnostic sensitivity (Sn) and specificity (Sp) of the iELISA based on mammalian cell-expressed N protein for detecting SARS-CoV-2-specific antibodies in cat serum samples.

**iELISA**	**Positive**	**Negative**	**Total**
			**(number)**
Positive (number)	44 **(Sn = 97.8%)**	1	45
Negative (number)	1	21 **(Sp = 95.5%)**	22
Predictive value	97.7%	95.5%	

**Figure 3:**
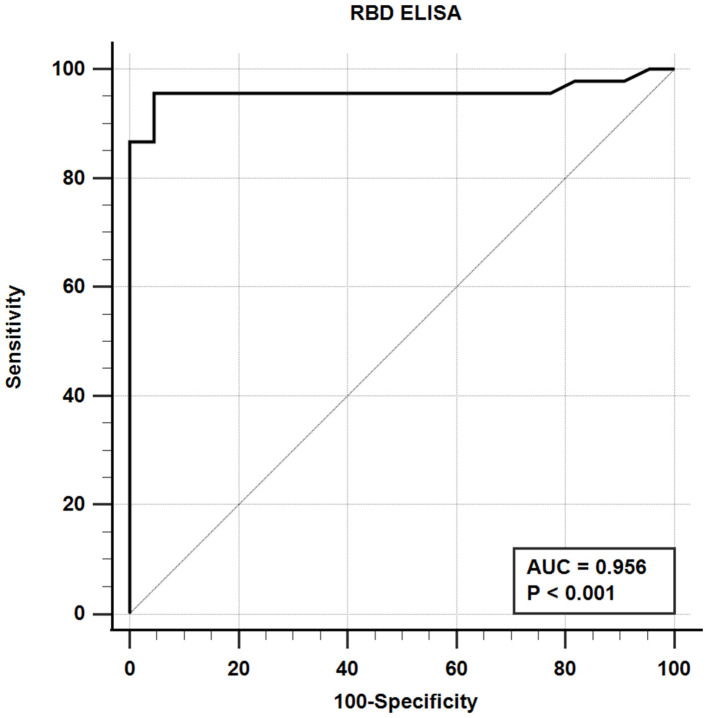
Receiver-operating characteristic (ROC) analysis for the RBD protein iELISA. The line represents the 95% confidence interval, and the upper left corner is the highest Youden index indicating the best combined sensitivity and specificity of 95.6% and 95.5%, respectively. AUC, area under the curve.

The iELISA using the recombinant mammalian-expressed RBD protein and well-characterized cat sera from experimentally SARS-CoV-2 virus-infected cats revealed that 21 out of 22 SARS-CoV-2-negative sera were negative and 43 out of 45 SARS-CoV-2-positive sera tested positive. Therefore, the RBD-specific iELISA has an estimated diagnostic sensitivity and specificity of 95.6 and 95.5%, respectively (Table 5; Figure 3).

**Table 5:** Diagnostic sensitivity (Sn) and specificity (Sp) of the RBD protein iELISA for detecting SARS-CoV-2-specific antibodies in the cat serum samples.

**iELISA**	**Positive**	**Negative**	**Total**
			**(number)**
Positive (number)	43 **(Sn = 95.6%)**	1	44
Negative (number)	2	21 **(Sp = 95.5%)**	23
Predictive value	97.7%	91.3%	

Two sera that were considered negative based on the VNT tested positive on the N or RBD iELISAs. Specifically, one negative serum (#026, 5 DPC) tested positive on both N iELISAs, and another negative serum (#328, 5 DPC) tested positive with the RBD iELISA. These sera were collected from experimentally infected cats at 5 DPC; therefore, it is possible that these animals started to seroconvert at 5 DPC. The high sensitivity of the iELISAs likely allowed for earlier detection of virus-specific antibodies in these samples compared with the VNT reference test.

**Figure 4:**
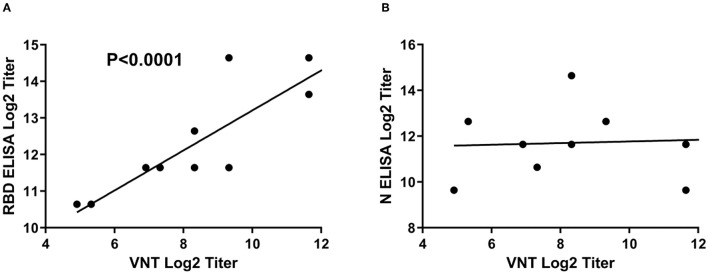
Correlation of SARS-CoV-2 iELISA antibody titers with virus-neutralizing antibody titers. Linear regression analysis of RBD **(A)** and N **(B)** iELISA antibody titers in relation to virus-neutralizing antibody titers using SARS-CoV-2-positive cat sera was performed using GraphPad Prism software; RBD r^2^ = 0.7924 and N r^2^ = 0.0041.

It is noteworthy to mention that there is a clear correlation between the RBD ELISA titers and the neutralization antibody titers as shown by the linear regression analysis in Figure 4. This analysis indicates that RBD iELISA titers correlate well with virus-neutralizing antibody titers (Figure 4A), whereas the N iELISAs show has no correlation (Figure 4B) with virus neutralization antibodies.

### Cross-Reactivity Between SARS-CoV-2 and Feline Coronaviruses

**Figure 5:**
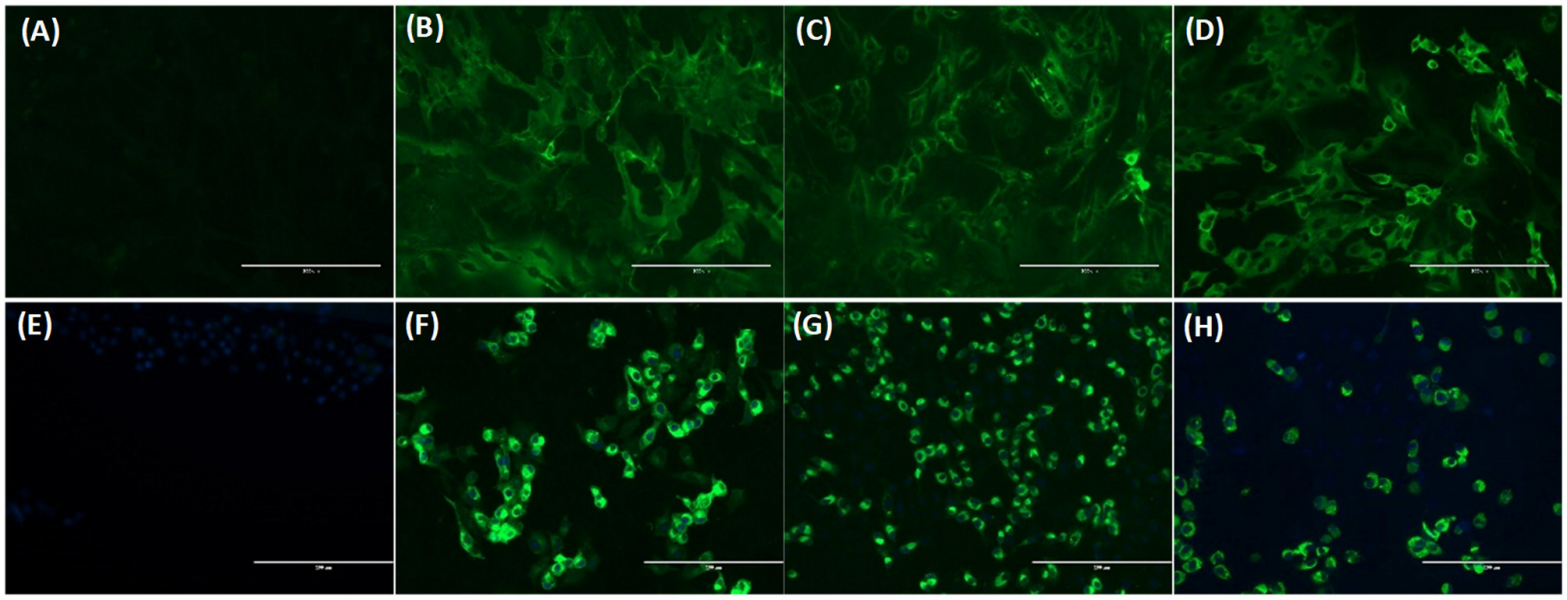
Immunofluorescence assay (IFA) analysis of Crandell feline kidney (CrFK) and Vero E6 cells infected with FeCoV virus **(A–D)** and SARS-CoV-2 **(E–H)**, respectively. Results of FeCoV IFA **(A–D)** with negative control cat serum **(A)**, FeCoV-positive cat serum #1 from Table 6 **(B)**, SARS-CoV-2-specific antibody-positive cat serum #328 from Table 7 **(C)**, and FeCoV N protein-specific monoclonal antibody **(D)**. IFA for SARS-CoV-2 **(E–H)** with FeCoV antibody-negative cat serum #12 from Table 6 **(E)**, SARS-CoV-2 antibody-positive cat serum #026 from Table 7 **(F)**, FeCoV antibody-positive serum #1 from Table 6 **(G)**, and SARS-CoV-2 RBD protein-specific monoclonal antibody #117A5 from Table 7 **(H)**. The photographs were taken using a fluorescence microscope for both IFAs with a serum dilution of 1:160.

FeCoV-positive and FeCoV-negative cat sera were analyzed to investigate the cross-reactivity of feline coronavirus-specific antibodies with SARS-CoV-2 antigens. A total of 19 cat sera (Table 2) representing 13 FeCoV-positive and 6 FeCoV-negative sera were tested using the three iELISAs coated with recombinant SARS-CoV-2 RBD and N proteins. The FeCoV-positive sera were clearly positive with the FeCoV-specific IFA test (titers >1:3,200), whereas the FeCoV-negative sera were negative in this test (Table 6). Nine out of 13 FeCoV-positive cat sera were also positive with at least one of the two N-specific iELISAs, and the N-iELISA-positive sera were also positive on SARS-CoV-2-infected cells (titers ranging from 1:80 to >1:2,400; Table 6). Importantly, these FeCoV-specific cross-reacting antibodies (SARS-CoV-2 N- and FeCoV-positive) did not react with the SARS-CoV-2 RBD region of the spike protein in the RBD-specific iELISA, and were also negative in a classical virus neutralization test with SARS-CoV-2. All FeCoV-negative serum samples were negative with the SARS-CoV-2 N and RBD antigens or in the SARS-CoV-2 IFA (Table 6; Figure 5).

**Table 6:** Cross-reactivity of FeCoV-specific antibodies with SARS-CoV-2.

**Cat ID**	**RBD**_**M**_		**N**_**M**_		**N**_**E**_		**VN**	**IFA (SARS-CoV-2)**		**IFA (FeCoV)**	
FeCoV pos #1	0.21	Neg	2.17	Pos	2.86	Pos	<1:20	>1:2,400	Pos	>1:6,400	Pos
FeCoV pos #2	0.15	Neg	1.29	Pos	1.32	Pos	<1:20	1:80	Pos	>1:6,400	Pos
FeCoV pos #0	0.18	Neg	1.89	Pos	1.53	Pos	<1:20	1:80	Pos	>1:6,400	Pos
FeCoV pos #3	0.09	Neg	0.2	Neg	0.28	Neg	<1:20	<1:40	Neg	>1:6,400	Pos
FeCoV pos #4	0.11	Neg	0.12	Neg	0.31	Neg	<1:20	<1:40	Neg	>1:6,400	Pos
FeCoV pos #5	0.31	Neg	0.26	Neg	0.37	Pos	<1:20	<1:40	Neg	1:3,200	Pos
FeCoV pos #6	0.12	Neg	0.19	Neg	0.53	Pos	<1:20	<1:40	Neg	>1:6,400	Pos
FeCoV pos #7	0.05	Neg	0.15	Neg	0.16	Neg	<1:20	<1:40	Neg	1:3,200	Pos
FeCoV pos #8	0.07	Neg	0.15	Neg	0.12	Neg	<1:20	<1:40	Neg	>1:6,400	Pos
FeCoV pos #27	0.09	Neg	2.63	Pos	1.74	Pos	<1:20	1:320	Pos	>1:6,400	Pos
FeCoV pos #28	0.28	Neg	2.03	Pos	2.41	Pos	<1:20	1:320	Pos	>1:6,400	Pos
FeCoV pos #29	0.07	Neg	0.97	Pos	1.29	Pos	<1:20	1:80	Pos	1:3,200	Pos
FeCoV pos #30	0.22	Neg	2.87	Pos	2.26	Pos	<1:20	>1:1,280	Pos	>1:6,400	Pos
FeCoV neg #9	0.23	Neg	0.09	Neg	0.55	Neg	<1:20	<1:40	Neg	<1:40	Neg
FeCoV neg #10	0.07	Neg	0.1	Neg	0.21	Neg	<1:20	<1:40	Neg	<1:40	Neg
FeCoV neg #11	0.27	Neg	0.1	Neg	0.24	Neg	<1:20	<1:40	Neg	<1:40	Neg
FeCoV neg #12	0.14	Neg	0.17	Neg	0.41	Neg	<1:20	<1:40	Neg	<1:40	Neg
FeCoV neg #14	0.09	Neg	0.1	Neg	0.1	Neg	<1:20	<1:40	Neg	<1:40	Neg
FeCoV neg #15	0.1	Neg	0.14	Neg	0.17	Neg	<1:20	<1:40	Neg	<1:40	Neg
FeCoV mAb	0.1	Neg	0.27	Neg	0.31	Neg	<1:20	<1:40	Neg	1:400	Pos

*FeCoV, feline coronavirus; RBD_M_, N_M_, mammalian cell-expressed RBD and N, respectively; N_E_, E. coli-expressed N; neg, negative; pos, positive; mAb, monoclonal antibody*.

Additional investigations into the cross-reactivity between FeCoV and SARS-CoV-2 were performed using: (i) a FeCoV N protein-specific monoclonal antibody (mAb), (ii) SARS-CoV-2 RBD-specific mAbs, and (iii) high titer SARS-CoV-2-positive cat sera. The FeCoV N-specific mAb was negative when used on SARS-CoV-2-infected cells by IFA and also negative in the virus neutralization test with SARS-CoV-2 (Table 6; Figure 5). The high-titer SARS-CoV-2 cat sera from the SARS-CoV-2 challenge study (20) and monoclonal antibodies against the RBD region of the SARS-CoV-2 spike protein were tested using the commercial FeCoV-specific IFA and by virus neutralization tests using infectious FeCoV. All four high-titer cat sera reacted positive in the FeCoV IFA, but negative in the virus neutralization test with FeCoV. In addition, the SARS-CoV-2 RBD-specific mAbs tested negative with the FeCoV IFA and in the virus neutralization test with FeCoV (Table 7). Both, the SARS-CoV-2-positive cat sera and the SARS-CoV-2 mAbs were positive in the SARS-CoV-2 IFA and the virus neutralization test (Table 7; Figure 5).

**Table 7:** Cross-reactivity of SARS-CoV-2-positive cat serum samples and SARS-CoV-2 RBD-specific monoclonal antibodies with FeCoV.

**Serum ID**	**IFA**		**VN**		**IFA**		**VN**	
	**(FeCoV)**		**(FeCoV)**		**(SARS-CoV-2)**		**(SARS-CoV-2)**	
#026 (21 DPC)	>1:40	Pos	<1:4	Neg	1:5,120	Pos	1:40	Pos
#328 (4 DP2C)	>1:320	Pos	<1:4	Neg	>1:5,120	Pos	1:160	Pos
#272 (4 DP2C)	>1:320	Pos	<1:4	Neg	>1:5,120	Pos	1:80	Pos
#903 (4 DP2C)	>1:320	Pos	<1:4	Neg	>1:5,120	Pos	1:160	Pos
mAb 91C2	<1:5	Neg	<1:4	Neg	1:1,280	Pos	1:32	Pos
mAb 91G12	<1:5	Neg	<1:4	Neg	1:2,560	Pos	1:64	Pos
mAb 117C1	<1:5	Neg	<1:4	Neg	1:640	Pos	1:256	Pos
mAb 117E2	<1:5	Neg	<1:4	Neg	1:2,560	Pos	1:64	Pos

*FeCoV, feline coronavirus; mAb, monoclonal antibody; DPC, days post-challenge; 4 DP2C, four days post-second challenge*.

## Discussion

The detection of SARS-CoV-2 virus RNA by RT-PCR is routinely used worldwide for the diagnosis of COVID-19 (12). However, reliable high-throughput serological assays to detect SARS-CoV-2-specific antibodies are needed to determine immune responses of different animal species to SARS-CoV-2 antigens (20); in addition, immune responses to vaccination or the presence of maternal antibodies in respective samples can also be measured (22, 30, 31). Serological tests indicate exposure to the virus or viral vaccine antigens (1, 13). Serological assays can facilitate prevalence studies or retrospective studies by detecting virus-specific antibodies in serum or other feasible samples.

The SARS-CoV-2 nucleocapsid (N) protein can be used as a target antigen due to the fact that it is an abundant, genetically highly conserved, and immunodominant protein of coronaviruses (7). In humans, N protein-specific antibodies can be detected as early as 8–14 days after infection (7, 32) which is earlier than spike (S) protein-specific human antibodies (7). Similarly, experimentally SARS-CoV-2-infected cats develop N-specific antibodies 5–7 days after virus challenge, but RBD-specific antibodies were detected only approximately 14 days after infection (20).

The S protein of SARS-CoV-2 is the major target for neutralizing antibodies which are critical for protective immunity against SARS-CoV-2 infections. The S1 subunit contains the RBD which is known to lack cross-reactivity between SARS-CoV-2 and SARS-CoV (24, 31, 33–36). Consequently, in the present study, we generated SARS-CoV-2-specific recombinant RBD and N proteins and used these proteins to develop indirect ELISA tests to detect antibodies in serum samples from cats experimentally infected with SARS-CoV-2. In-house SARS-CoV-2 ELISA tests for analysis of human sera were developed by different research groups, with sensitivity and specificity between 73.7 and 99.3% and 91.7 and 100%, respectively (37). Sensitivities of our in-house ELISA tests using the RBD (expressed in mammalian cells), N_*E*_ (expressed in *E. coli*), and N_*M*_ (expressed in mammalian cells) antigens were 95.6, 93.3, and 97.8% and the specificities were 95.5, 95.5, and 95.5%, respectively. The N_M_ antigen-coated ELISA was more sensitive compared with the RBD iELISA in our study; these data correlate with results obtained by Burelo et al. (7). Interestingly, both N antigen iELISAs, one based on the recombinant N protein expressed in *E. coli* and the other in mammalian cells, had similar sensitivities and specificities when tested with positive and negative cat sera (Tables 3, 4). This indicates that both prokaryotic and eukaryotic expression systems can be used to produce recombinant N protein as antigens for a SARS-CoV-2 ELISA. Our study also indicates that the N_*M*_ and N_*E*_ protein-based iELISAs is more sensitive than the RBD protein-based iELISA to detect SARS-CoV-2-specific IgG antibodies in cat serum. However, some FeCoV-positive cat sera cross-react with the SARS-CoV-2 N-based iELISAs as shown in this study (Table 6). Other studies also identified cross-reactivity between the N protein of SARS-CoV-2 and the N protein of other human betacoronaviruses (38–41), but none of these studies investigated cross-reactivity between the SARS-CoV-2 N protein and alphacoronavirus-specific antibodies as done here. In addition, SARS-CoV-2-positive cat sera (infected with a Wuhan-like virus) reacted with FeCoV-infected cells using an IFA-based commercial test system (Table 7). However, there was no cross-reactivity of FeCoV-positive cat sera with the SARS-CoV-2 RBD antigen (Table 7), and vice versa of SARS-CoV-2 RBD-specific mAb with FeCoV-infected cells in the IFA test (Table 6). Importantly, high titer SARS-CoV-2-specific cat sera did not neutralize FeCoV (Table 7), nor did FeCoV-positive cat sera neutralize SARS-CoV-2 (Table 6). Since FeCoV-positive sera reacted with SARS-CoV-2 N, but not RBD antigen (see Table 6), and SARS-CoV-2-specific hyperimmune sera, but not RBD-specific monoclonal antibodies cross-reacted with FeCoV (see Table 7), we conclude that the cross-activity is most likely based on the N and/or other SARS-CoV-2 antigens, but not on the SARS-CoV-2 RBD antigen.

In summary, iELISA tests based on recombinant N and RBD proteins of SARS-CoV-2 were developed and optimal conditions for the iELISAs were established. The diagnostic sensitivity for the detection of feline antibodies specific for the SARS-CoV-2 N or RBD proteins of the iELISA tests was between 93.3 and 97.8% vs. 95.6%, respectively, and the diagnostic specificity was 95% for all three tests. In addition, we found a clear correlation between SARS-CoV-2 RBD-specific antibody and virus-neutralizing antibody titers. In conclusion, the iELISAs described here can be used for high-throughput screening of cat sera for the presence of SARS-CoV-2-specific antibodies in a BSL-2 biocontainment environment, as opposed to virus neutralization tests with live virus which require a BSL-3 laboratory.

## Data Availability Statement

The original contributions presented in the study are included in the article/supplementary material, further inquiries can be directed to the corresponding author/s.

## Author Contributions

JAR, RP, and BZ were involved in the study conception and design. DB, GR-S, and NG were involved in the data collection, analysis, and interpretation of results. DB prepared the first draft of the manuscript. All authors revised and reviewed the manuscript and agree to be accountable for the content of the work.

## Funding

Funding for this study was partially provided through Grants from the National Bio and Agro-Defense Facility (NBAF) Transition Fund from the State of Kansas (JAR), the AMP Core of the Center of Emerging and Zoonotic Infectious Diseases (CEZID) from the National Institute of General Medical Sciences (NIGMS) under Award Number P20GM130448, the Department of Homeland Security Center of Excellence for Emerging and Zoonotic Animal Diseases under Grant Number HSHQDC 16-A-B0006, the NIAID Centers of Excellence for Influenza Research and Surveillance under Contract Number HHSN 272201400006C, and the NIAID supported Centers of Excellence for Influenza Research and Response (CEIRR, Contract Number 75N93021C00016), the Food and Drug Administration Veterinary Laboratory Investigation and Response Network, and the National Institute of Health Award Number U18FD007509-01.

## Author Disclaimer

Mention of trade names or commercial products in this publication is solely for the purpose of providing specific information and does not imply recommendation or endorsement by Kansas State University.

## Conflict of Interest

JAR laboratory received support from Tonix Pharmaceuticals, Xing Technologies, Genus plc, and Zoetis, outside of the reported work. JAR is inventor on patents and patent applications on the use of antivirals and vaccines for the treatment and prevention of virus infections, owned by Kansas State University, KS. The remaining authors declare that the research was conducted in the absence of any commercial or financial relationships that could be construed as a potential conflict of interest.

## Publisher's Note

All claims expressed in this article are solely those of the authors and do not necessarily represent those of their affiliated organizations, or those of the publisher, the editors and the reviewers. Any product that may be evaluated in this article, or claim that may be made by its manufacturer, is not guaranteed or endorsed by the publisher.
